# Supplementary material for: Spinal pain patients seeking care in primary care and referred to physiotherapy: A cross-sectional study on patients characteristics, referral information and physiotherapy care offered by general practitioners and physiotherapists in France
Source: PLoS One. 2022 Sep 6;17(9):e0274021. doi: 10.1371/journal.pone.0274021 (PMC9447922; doi:10.1371/journal.pone.0274021)
Supplement: S1 File — (ZIP) [file pone.0274021.s001.zip › Supporting information - S2 Table.docx]

| **Section** | **Item #** | **Checklist item** | **Reported on page #** |
| --- | --- | --- | --- |
| Title/Abstract | 1 | Identify in title or abstract that interrater/intrarater  reliability or agreement was investigated. | 2 |
| Introduction | 2 | Name and describe the diagnostic or measurement device of interest explicitly. | 3,4 |
|  | 3 | Specify the subject population of interest. | 3,4 |
|  | 4 | Specify the rater population of interest (if applicable). | 3,4 |
|  | 5 | Describe what is already known about reliability and  agreement and provide a rationale for the study (if applicable). | 3 |
| Methods | 6 | Explain how the sample size was chosen. State the determined number of raters, subjects/objects, and replicate observations. | 4,5 |
|  | 7 | Describe the sampling method. | 4,5 |
|  | 8 | Describe the measurement/rating process (e.g. time interval between repeated measurements, availability  of clinical information, blinding). | 7 |
|  | 9 | State whether measurements/ratings were conducted independently. | 7,8 |
|  | 10 | Describe the statistical analysis. | 7,8 |
| Results | 11 | State the actual number of raters and subjects/objects  which were included and the number of replicate observations which were conducted. | 19,20 |
|  | 12 | Describe the sample characteristics of raters and  subjects (e.g. training, experience). | 19,20 |
|  | 13 | Report estimates of reliability and agreement including measures of statistical uncertainty. | 19,20 |
| Discussion | 14 | Discuss the practical relevance of results. | 24 |
| Auxiliary material | 15 | Provide detailed results if possible (e.g. online). |  |

**S2 Table**. Reporting items of the Guidelines for reporting reliability and agreement studies (GRRAS)

*Version based on: Kottner J, Audigé L, Brorson S, Donner A, Gajeweski BJ, Hróbjartsson A, Robersts C, Shoukri M, Streiner DL. Guidelines for reporting reliability and agreement studies (GRRAS) were proposed. J Clin Epidemiol. 2011;64(1):96-106*
