# Supplementary material for: Spinal pain patients seeking care in primary care and referred to physiotherapy: A cross-sectional study on patients characteristics, referral information and physiotherapy care offered by general practitioners and physiotherapists in France
Source: PLoS One. 2022 Sep 6;17(9):e0274021. doi: 10.1371/journal.pone.0274021 (PMC9447922; doi:10.1371/journal.pone.0274021)
Supplement: S1 File — (ZIP) [file pone.0274021.s001.zip › Supporting information - S3 Table.docx]

|  | **Strength of evidence for the physiotherapy management of spinal pain patients** | | | |
| --- | --- | --- | --- | --- |
|  | **Neck pain [25,26]** | **Thoracic spine pain [26,27]** | **Low back pain [23,24]** | **Combination of spinal pain [23-27]** |
| Postural and hygenic education^†^ | **Limited evidence** | **Limited evidence** | **Limited evidence** | **Limited evidence** |
| Specific spinal exercises^‡^ | **Strong evidence** | **Strong evidence** | **Strong evidence** | **Strong evidence** |
| McKenzie exercises | **Moderate evidence** | **Limited evidence** | **Limited evidence** | **Limited evidence** |
| Stretching exercises | **Limited evidence** | **Limited evidence** | **Limited evidence** | **Limited evidence** |
| General exercises^¤^ | **Moderate evidence** | **Moderate evidence** | **Moderate evidence** | **Moderate evidence** |
| Manual therapy^§^ | **Limited evidence** | **Limited evidence** | **Limited evidence** | **Limited evidence** |
| Massage therapy | **Limited evidence** | **Limited evidence** | **Limited evidence** | **Limited evidence** |
| Hot/Cold therapy | **Limited evidence** | **Limited evidence** | **Limited evidence** | **Limited evidence** |
| Electrotherapy | **Limited evidence** | **Limited evidence** | **Limited evidence** | **Limited evidence** |
| Ultrasound therapy | **Insufficient** | **Insufficient** | **Insufficient** | **Insufficient** |

**S3 Table.** Strength of evidence categorized as strong, moderate, limited or insufficient based on evidence from clinical practice guidelines and systematic overviews selected for the physiotherapy management of spinal pain patients [23-27]

*Recommendations extracted were based from one French CPG on the management of low back pain, and two systematic overviews of current evidence for the management of neck pain [25,26], and two on thoracic spine pain [26,27]*

*^†^ Such as postural hygiene or advice on daily physical activity*

*^‡^Defined as coordination, endurance, strengthening or postural exercises.*

*^¤^Defined as primarily range of motion and strengthening exercise of the whole body.*

*^§^Defined as spinal joints mobilization or manipulation and neurodynamic technique primarily tailored range of motion.*
