# Supplementary material for: Spinal pain patients seeking care in primary care and referred to physiotherapy: A cross-sectional study on patients characteristics, referral information and physiotherapy care offered by general practitioners and physiotherapists in France
Source: PLoS One. 2022 Sep 6;17(9):e0274021. doi: 10.1371/journal.pone.0274021 (PMC9447922; doi:10.1371/journal.pone.0274021)
Supplement: S1 File — (ZIP) [file pone.0274021.s001.zip › Supporting information - S4 Table.docx]

|  | **Neck pain  (n=20)  *n (%)*** | **Thoracic spine pain (n=6)  *n (%)*** | **Low back pain (n=66) *n (%)*** | **Combination of spinal pain  (n=44) *n (%)*** | **All patients  (n=136)  *n (%)*** |
| --- | --- | --- | --- | --- | --- |
| Postural and hygenic education^†^ | 7 (35.0) | 1 (16.7) | 25 (37.9) | 12 (27.3) | 45 (33.1) |
| Specific spinal exercises^‡^ | 12 (60.0) | 3 (50.0) | 41 (62.1) | 24 (54.5) | 80 (58.8) |
| McKenzie exercises | 4 (20.0) | 1 (16.7) | 13 (19.7) | 5 (11.4) | 23 (16.9) |
| Stretching exercises | 4 (20.0) | 1 (16.7) | 25 (37.9) | 16 (36.4) | 46 (33.8) |
| General exercises^¤^ | 2 (10.0) | 0 (0.0) | 7 (10.6) | 0 (0.0) | 9 (6.6) |
| Manual therapy^§^ | 11 (55.0) | 4 (66.7) | 32 (48.5) | 0 (0.0) | 47 (34.6) |
| Massage therapy | 6 (30.0) | 2 (33.3) | 25 (37.9) | 17 (38.6) | 50 (36.8) |
| Hot/Cold therapy | 1 (5.0) | 0 (0.0) | 5 (7.6) | 3 (6.8) | 9 (6.6) |
| Electrotherapy | 0 (0.0) | 0 (0.0) | 4 (6.1) | 1 (2.3) | 5 (3.7) |
| Ultrasound therapy | 0 (0.0) | 0 (0.0) | 0 (0.0) | 0 (0.0) | 0 (0.0) |

**S4 Table.** Description of specific physiotherapy interventions recommended by physiotherapists at their initial consultation to spinal pain patients for whom no specific physiotherapy interventions were prescribed by general practitioners (n=136)

*Physiotherapy interventions categories based on clinical practice guidelines and systematic reviews selected [23-27]*

*^†^ Such as postural hygiene or advice on daily physical activity*

*^‡^Defined as coordination, endurance, strengthening or postural exercises.*

*^¤^Defined as primarily range of motion and strengthening exercise of the whole body.*

*^§^Defined as spinal joints mobilization or manipulation and neurodynamic technique primarily tailored range of motion.*
