# Supplementary material for: Spinal pain patients seeking care in primary care and referred to physiotherapy: A cross-sectional study on patients characteristics, referral information and physiotherapy care offered by general practitioners and physiotherapists in France
Source: PLoS One. 2022 Sep 6;17(9):e0274021. doi: 10.1371/journal.pone.0274021 (PMC9447922; doi:10.1371/journal.pone.0274021)
Supplement: Attachment — Submitted filename: Response to Reviewers.docx [file pone.0274021.s003.docx]

**Response to comments from the editor and reviewers - Manuscript PONE-D-21-38018R1**

We appreciate the time invested by the Editor and reviewers for their thoughtful comments and for the opportunity given to improve our manuscript. We provide below the detailed answers to each comment.

**Academic Editor:**

1. **The rationale of the study should be explained in detail. You should focus on three elements of introduction:**
   a. **What is known about the topic? (Background)**
   b. **What is not known? (The research problem)**
   c. **Why the study was done? (Justification)**

We have completed and reorganized the introduction by cutting it as suggested:

- Background (p2, lines 29-52);
- The research problem (p2-3, lines 52-57);
- Justification (p3, lines 58-61).

The introduction now quotes :

Background :

« Musculoskeletal disorders (MSKDs) are a major public health concern worldwide and represent globally the second most important group of disorders in terms of years lived with disability (1). The most common MSKDs encountered in primary care are spinal pain including neck, thoracic spine or low back disorders (2,3). For the majority of patients consulting for spinal pain in primary care, there is strong evidence supporting the benefit of early physiotherapy in the care pathway for these patients (4,5). However, this early access is not always carried out as systematically and smoothly as intended. The consequences are delayed treatment and potential clinical complications such as chronic pain (6–8).

Depending on the country, access to primary care is organized or constrained in different ways. In some health care systems, general practitioners (GPs) are first-contact providers for patients seeking care for spinal pain complaints. GPs have a key role in the patient’s care pathway by providing an initial diagnosis and treatment and referring to other appropriate health care professionals such as physiotherapists (PTs) (9). The purpose of this referral is to validate the indication for physiotherapy care and to identify any contraindications or precautions to rehabilitation for a specific patient (9). However, several studies have concluded that diagnoses provided by GPs for this population may often be erroneous or not as accurate as those provided by other MSKD specialists such as sports physicians, orthopedic surgeons or even PTs (10–12). In addition, GPs’ practice patterns in terms of treatment recommendations have been reported to divert significantly from established evidence-based clinical practice guidelines (CPGs); poor patient education as well as poor promotion of active treatments such as physical activity is often reported (6,7,13–18). An Australian study showed that the percentage of patients seeing a GP for MSKDs who were referred to physiotherapy was low out of the total number of patients seen by the physician with the same condition (19). An observational study in Denmark reported that, for patients with various MSKDs, GPs' primary diagnoses were generally poorly defined with the use of vague terms such as myopain or back-related diagnoses (20).

France belongs to those countries where the GP is the gatekeeper to the health care system and might secondarily refer a patient with MSKDs to a PT. MSKDs account for approximately 17.0% of French GPs consultations (13,21) and their incidence is expected to increase as the French population is ageing rapidly (22). »

Research problem :

“French GPs’ physiotherapy referral practices for patients with spinal pain complaints in primary care have not been described and reported so far. Thus, the extent to which French GPs and PTs practices as regards to physiotherapy interventions prescribed, are supported by evidence-based recommendations of CPGs is not known. Moreover, to our knowledge, no study has been conducted to compare patient’s MSK diagnostic and physiotherapy interventions concordance between GPs and PTs with respect to CPGs.”

Justification :

“In a system where the GP is the patient's first access, even if it is expected that his/her knowledge of evidence-based practice is up to date, it is likely that this knowledge cannot be exhaustive on such a specific subject, due to the polyvalence of this specialty. Similarly, it is expected that the skills acquired by the PT profession are at a high level of expertise and therefore in line with CPGs.”

2. **Add a clear hypothesis of the study.**

An hypothesis has been added (p3, lines 61-64) :

“This study assumes that the characteristics of referral to physiotherapy described by French GPs for patients with spinal pain are incompletely reported, as in other countries, and that physiotherapy interventions recommended by PTs could be significantly more consistent with CPGs than those prescribed by GPs.”
